# Epigenetic Regulators of White Adipocyte Browning

**DOI:** 10.3390/epigenomes5010003

**Published:** 2021-01-12

**Authors:** Ravikanth Nanduri

**Affiliations:** Laboratory of Metabolism, Center for Cancer Research, National Cancer Institute, National Institutes of Health, Bethesda, MD 20892, USA; ravikanth.nanduri@nih.gov

**Keywords:** white adipocytes, WAT browning, beige adipocytes, epigenetic regulators

## Abstract

Adipocytes play an essential role in maintaining energy homeostasis in mammals. The primary function of white adipose tissue (WAT) is to store energy; for brown adipose tissue (BAT), primary function is to release fats in the form of heat. Dysfunctional or excess WAT can induce metabolic disorders such as dyslipidemia, obesity, and diabetes. Preadipocytes or adipocytes from WAT possess sufficient plasticity as they can transdifferentiate into brown-like beige adipocytes. Studies in both humans and rodents showed that brown and beige adipocytes could improve metabolic health and protect from metabolic disorders. Brown fat requires activation via exposure to cold or β-adrenergic receptor (β-AR) agonists to protect from hypothermia. Considering the fact that the usage of β-AR agonists is still in question with their associated side effects, selective induction of WAT browning is therapeutically important instead of activating of BAT. Hence, a better understanding of the molecular mechanisms governing white adipocyte browning is vital. At the same time, it is also essential to understand the factors that define white adipocyte identity and inhibit white adipocyte browning. This literature review is a comprehensive and focused update on the epigenetic regulators crucial for differentiation and browning of white adipocytes.

## 1. Introduction

Coordinated regulation of food intake and energy expenditure are essential for the survival of all living organisms. In mammals, adipocytes play a central role in maintaining energy homeostasis. There are three types of adipocytes found in humans and rodents—white, brown, and beige adipocytes. White adipocytes consisting of a single large lipid droplet and are specialized to store excess energy in the form of triglycerides when nutrients are surplus [1]. At the same time, in nutrient-deficient conditions, white adipocytes ensure rapid lipid mobilization and supply energy to all other organs in the form of free fatty acids [2]. Furthermore, white adipose tissue (WAT) also communicates with other metabolic organs by secreting various adipokines, thus contributing towards systemic energy homeostasis [3]. In addition, excessive accumulation of WAT is a hallmark of obesity and increases the risk of type II diabetes, hyperlipidemia, hypertension, and cardiovascular diseases [4,5].

Unlike white adipocytes, brown adipocytes are specialized to burn fat and produce heat. Beige adipocytes also can burn fat and are found as clusters within the WAT. Both brown and beige adipocytes can produce heat and protect mammals from hypothermia. Heat production occurs through uncoupling protein 1 (UCP1), present in the inner mitochondrial membrane, which delinks ATP synthesis and oxidative phosphorylation. Cold exposure activates classical brown adipocytes and also induces beige adipocyte formation [6]. Mechanistically, cold exposure activates the sympathetic nervous system and results in the release of norepinephrine, which can bind to β-adrenergic receptors (β-ARs), leading to the induction of UCP1 protein [7,8]. Although all three adipocyte cell types originate from mesenchymal stem cells (MSCs), brown adipocytes are differentiated from *Myf5 positive* precursor cells and white adipocytes are differentiated from *Myf5 negative *precursors [9]. Notably, recent reports also suggested the presence of unique beige preadipocytes in WAT [10].

Regardless of adipocyte type, adipogenesis is a complicated process of epigenetic changes, which involves significant changes to the chromatin. The epigenetic changes during adipocyte differentiation are attributed to histone-modifying enzymes, DNA methylases, transcription factors (TFs), and microRNAs (miRNA). Both white and thermogenic fat cells possess unique epigenetic regulators, despite having many in common such as Peroxisome proliferator-activated receptors (PPARs) and CCAAT/enhancer-binding proteins (CEBPs) [11].

**Figure 1:**
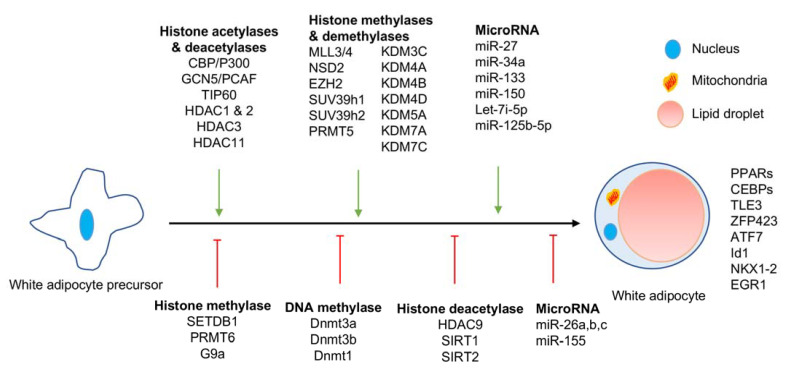
Epigenetic regulators of white adipocyte differentiation.

**Figure 2:**
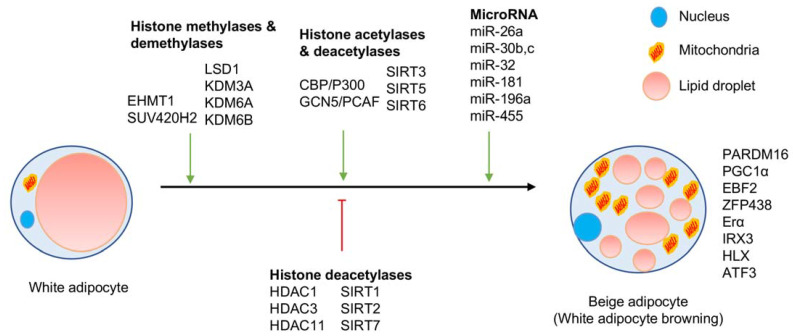
Epigenetic regulators of white adipocytes browning.

Cold or β-AR agonists can activate BAT and induce mature white adipocyte conversion to beige adipocytes [12]. However, prolonged exposure to cold is not therapeutically relevant and the usage of β-AR agonists for therapeutic purposes in treating dyslipidemia and obesity is still in question with their associated side effects [13,14]. Hence, in identifying potential therapeutic targets, it is essential to focus on different epigenetic regulators associated with white adipocyte differentiation and their browning. Epigenetic regulators that are reported to alter white adipocyte differentiation are summarized in Figure 1, while epigenetic regulators that modulate the browning of white adipocytes are represented in Figure 2.

## 2. Histone Acetyltransferases (HATs)

Histone acetylation is one of the first histone modifications discovered to influence transcription. Acetylation adds negative charge to the histones’ lysine residue, which repels negatively charged DNA, resulting in a decompaction of chromatin structure [15]. These acetylation reactions are catalyzed by histone acetyltransferases (HATs) and contribute to gene activation. HATs are categorized into three major families based on the mechanism that they transfer acetyl group: the CREB binding protein (CBP)/P300 family (CBP, P300), the GCN5-related N-acetyltransferases (GNAT) family (GCN5, PCAF, and Hat1, etc.), and the MYST family (MYST1, MYST2, TIP60, etc.) [16]. Other HATs reported are transcriptional coactivators, such as steroid receptor coactivator 1 (SRC1) and Transcriptional intermediary factor-2 (TIF2). The role of histone modifications in adipogenesis was first characterized in mouse white preadipocyte cell line 3T3L-1, in which Histone H3 acetylated at lysine 27 acetylation (H3K27ac) was associated with most active chromatin and displayed dynamic distribution on the promoter, enhancer, intronic, and intergenic regions [17]. However, not all H3K27ac regions overlapped with active chromatin [18]. H3K27ac was also highly induced during preadipocyte differentiation and correlated with *Peroxisome proliferator-activated receptor γ (Pparγ)* gene expression, a master TF for adipogenesis [17,19]. 

CBP and P300 are a functionally redundant HAT pair reported to acetylate H3K27 and are reported to be indispensable for adipocyte differentiation via activation of PPARγ [20,21]. Both these HATs’ expressions are induced during differentiation [22]. The reduced WAT level in *cbp* heterozygous mice suggests CBP’s critical role in white adipocyte differentiation [23]. HATs also define how environmental factors define cell fate, i.e., cold-induced beige adipocytes displayed an H3K27ac pattern like brown adipocytes, while warming of beige adipocytes induced an H3K27ac pattern similar to white adipocytes, indicating the crucial role these HATs play in defining adipocyte identity [12].

GCN5 and PCAF (P300/CBP-associated factor) are HATs that have been shown to acetylate Histone H3 acetylated at lysine 9 (H3K9ac) [20]. Besides this, HATs acetylate CEBPβ (CCAAT/enhancer-binding protein β) to promote adipocyte differentiation. In brown preadipocytes, the loss of GCN5/PCAF inhibited adipogenesis by suppressing PPARγ and PR domain containing 16 (PRDM16) expression and indicates the probable role of H3K9 acetylation in WAT browning [24]. ACE2 (angiotensin-converting enzyme 2), known to possess anti-obesity effects when injected into high-fat diet (HFD)-fed mice, enhanced H3K9ac in WAT, indirectly suggesting the crucial role played by GCN5/PCAF in WAT browning [25].

HIV-1 Tat interacting protein 60 (TIP60) is a novel positive regulator of PPARγ and adipogenesis [26]. Mass spectrometry and chromatin immunoprecipitation (ChIP) experiments revealed that TIP60 protein interacts with PPARγ and is recruited to the PPARγ target genes in mature 3T3L-1 adipocytes. Other HATs are reported to alter adipogenesis, but their role is attributed to target proteins’ acetylation instead of histone acetylation. Transcriptional intermediary factor-2 (TIF2) knockout mice were protected from obesity with increased energy expenditure, whereas steroid receptor coactivator 1 (SRC1) knockout mice are prone to obesity due to lowered energy expenditure [27]. The absence of TIF2 abrogated PPARγ activity and displayed smaller white adipocytes, imitating WAT browning. In summary, most of the HATs reported until now positively regulate adipogenesis and this effect was attributed to both histone acetylation and target protein acetylation.

## 3. Histone Deacetylases (HDACs)

Histone deacetylation reactions are catalyzed by histone deacetylases (HDACs), which imparts gene repression. HDACs are classified into four different classes: class I (HDAC1–3 and 8), class IIa (HDAC4, 5, 7, and 9), class IIb (HDAC6 and 10), class III (SIRT1–7), and class IV (HDAC11) [16]. Investigations on HDACs in adipocytes further highlighted the importance of histone acetylation in this process. Several reports showed that HADC1, HADC2, HDAC3, HDAC9, and HDAC11 are associated with the deacetylation of H3K27ac [28,29,30,31,32]. As HATs promote adipocyte differentiation and browning, HDACs are assumed to inhibit adipocyte differentiation. Conversely, mouse embryonic fibroblasts (MEF) from *Hdac1* and *Hdac2* knockout mice could not differentiate into adipocytes induced by a hormone cocktail of isobutylmethylxanthine, dexamethasone, insulin, and rosiglitazone, while deletion of individual HDAC did not have any affect [33]. Treatment with deacetylase inhibitor trichostatin A also inhibited PPARγ expression and adipogenesis in 3T3L-1 preadipocytes. HDAC1 protein levels were enriched in WAT and negatively correlated with thermogenic genes *Ucp1* and *Pparγ coactivator 1α* (*Pgc1α*) expression [29]. Similarly, treatment with class I HDAC inhibitors SAHA and MS275 increased brown adipocyte-specific genes such as *Ucp3*, *Pparγ*, *Prdm16*, *Adrb3*, and *Ucp1* in WAT of obese mice, suggesting a negative correlation of HDACs with WAT browning [34]. Mechanistically, these inhibitors were shown to reduce HDAC3 recruitment to the *pgc1α* gene promoter. Selective deletion of HDAC3 in adipose tissue induces H3K27ac on enhancers of *Pparγ*, *Ucp1*, and regulatory regions of *Pparα*, thereby promoting WAT’s oxidative capacity and browning [31]. HDAC11 also mediates the repression of brown adipocyte differentiation and WAT browning through its interaction with Bromodomain-containing protein 2 (BRD2), and the deletion of HDAC11 induces WAT’s browning in mice fed an HFD [32]. Contrarily, HDAC9 has been shown to negatively regulate adipogenesis by inhibiting the expression of *Cebpα* through directly binding to its promoter [35]. Preadipocytes from HDAC9^−/−^ mice displayed increased adipogenesis, while overexpression of HDAC9 in 3T3L-1 cells abrogated adipogenesis. It has also been reported that HFD-induced defects in adipocyte differentiation are also associated with elevated HDAC9 expression, as its deletions improved the metabolic state of the HFD-fed mice [36].

Among the SIRT family of histone deacetylases, SIRT1, SIRT2, SIRT3, SIRT5, and SIRT6 are reported to deacetylate H3K9ac [37,38,39,40]. SIRT1 deacetylates the *Sfrp1*, *Sfrp2*, and *Dact1* promoters at H3K9 and H4K16, which activates Wnt signaling, inhibiting adipocyte differentiation of MSCs [41]. Notably, mice with adipocyte-specific deletion (both white and brown) of SIRT1 displayed increased glucose tolerance, insulin sensitivity, and decreased inflammation compared to control mice [42]. Moreover, SIRT1 KO mice also displayed decreased expression of *Ucp1*, *Fibroblast growth factor 21* (*fgf21)*, *Pgc1α*, and *Cox7a* in its epididymal WAT (eWAT) upon exposure to HFD. Interestingly, SIRT1 has been shown to promote WAT browning by deacetylating PPARγ [43]. Furthermore, SIRT2 inhibits PPARγ, thereby adipogenesis in 3T3L-1 cells by promoting interaction of FOXO1 and PPARγ [44]. In contrast, shRNA-mediated depletion of SIRT3 abrogated adipocyte differentiation of adipose-derived MSC [45]. Additionally, SIRT3 upregulation during early adipocyte differentiation is essential for mitochondrial biogenesis and function [46]. SIRT3 is also shown to increase UCP1 expression and localize in BAT’s inner mitochondrial membrane [47]. Loss of another mitochondrial sirtuin, SIRT4, led to decreased expression of critical adipogenic genes and inhibited bovine adipocyte differentiation [48]. The knockdown of SIRT5 contributed to decreased intracellular α-ketoglutarate concentration in mice, which led to elevated H3K9me2/3 on *Pparγ* and *Prdm16* promoters and inhibited WAT browning [49]. This effect of SIRT5 on H3K9me2/3 could be due to a decrease in H3K9 acetylation. Mice with adipocyte-specific deletion of SIRT6 displayed increased body weight, fat mass, and impaired metabolic homeostasis [50]. SIRT6 has also been shown to promote adipogenesis by inhibiting mitotic clonal expansion by suppressing Kinesin Family Member 5C (KIF5C) expression [51]. Loss of SIRT6 additionally inhibited WAT browning following cold exposure or β3-AR agonist treatment. SIRT7^−/−^ mice displayed reduced WAT mass, and this defect was imputed to increased SIRT1 activity [52]. SIRT7 deacetylates SIRT1 and prevents SIRT1′s ability to deacetylate H3K9 and H4K16. In summary, HDAC1-3, HDAC11, SIRT3, SIRT5, and SIRT6 are crucial for WAT browning, while HDAC9, SIRT1, SIRT2, and SIRT7 are inhibiting WAT browning (Figure 2). This bi-directional regulation of histone deacetylases might be due to histone deacetylation of specific target gene promoters or by deacetylating their target proteins. 

## 4. Histone Methyltransferases (HMT)

Histone methylation is another significant modification on histones that regulates the functional state of the chromatin. The histone methylation reactions are catalyzed by HMTs [53]. Depending on the residue and valency, methylation can define active and inactive chromatin states. Based on residue, HMTs are two types—lysine methyltransferases (KMTs) and arginine methyltransferases (RMTs). Methylation of histone H3 on K4, K36, and K79 correlates with gene activation [54]. Trimethylation on H3K4 (H3K4me3) marks for promoters while di or monomethylation of H3K4 (H3K4me1/2) marks enhancers of actively transcribed genes. Di and trimethylation of H3K9 and H3K27 (H3K9me2, H3K9me3, H3K27me2, and H3K27me3) are considered as repressive marks, whereas monomethylation on these residues (H3K9me1, H3K27me1) marks for active chromatin [55,56]. Notably, repressive methylation of H3K9 recruits heterochromatin-associated protein-1 (HP-1) to establish heterochromatin [57], whereas repressive methylation of H3K27 is involved in polycomb group-mediated gene silencing [58]. Several other histone residues are also reported to be methylated and modulate gene function and are discussed elsewhere [56].

Mice expressing an inactivated mutant of H3K4-specific methyltransferase mixed-lineage leukemia protein 3 (MLL3/KMT2C) had markedly reduced white fat but BAT amount stayed the same. Additionally, MEF cells from these mutant mice are less responsive to adipogenesis inducers, suggesting that MLL3 plays a vital role in WAT physiology. RNA seq analyses of WAT and BAT from this MLL3 mutant mice displayed alteration in their gene expression pattern associated with metabolism [59]. Notably, these mice weighed 20% less than control mice and displayed increased energy expenditure, and insulin sensitivity. Mixed-lineage leukemia protein 4 (MLL4/KMT2B) possesses partial functional redundancy with its paralog MLL3. Studies using conditional knockout of MLL4 revealed that MLL4 was recruited along with lineage-determining TFs during adipogenesis, and its loss significantly decreased H3K4me1/2 and H3K27ac in cells, indicating MLL4 is vital for adipogenesis [60]. H3.3K4M mutant mice or MLL3/4 SET domain deleted mice displayed impaired adipogenesis in brown preadipocytes, suggesting the redundant role of these proteins in adipocytes’ browning [61]. Importantly, MLL3/4 is required for CBP/p300 binding to enhancers and for super-enhancer formation during adipogenesis [62]. Furthermore, MLL3/4-associated proteins PAX-interacting protein 1 (PTIP) and PAXIP1 Associated Glutamate Rich Protein 1 (PAGR1) are shown to regulate the expression of CEBPβ, CEBPδ, CEBPα, and PPARγ during adipogenesis [63,64].

Studies on the adipose-specific deletion of H3K9me^2^/me^3^ methyltransferase euchromatic histone-lysine N-methyltransferase 1 (EHMT1/KMT1D) in mice revealed that the loss of EHMT1 severely abrogated thermogenesis in WAT and BAT [65]. EHMT1′s ability to induce WAT browning and BAT was attributed to its interaction with PRDM16, a coregulator of PPARγ. In contrast, the deletion of H3K9me2 methyltransferase G9A (KMT1C) enhances C/EBPβ binding to *Pparγ* promoter and enhances adipogenesis [66]. Genome-wide studies revealed the occupancy of G9a and H3K9me2 on the *pparγ* locus. Another H3K9-specific methyltransferase SET domain bifurcated histone lysine methyltransferase 1 (SETDB1/KMT1E) has been reported to trimethylate H3K9 near the transcription start site which is previously marked with H3K4me3 to establish bivalent modification to PPARγ and CEBPα [67]. This bivalent modification is essential for MSCs and preadipocytes’ commitment to adipogenesis to maintain master TF genes to be expressed at low levels and keep them paused for activation when required for differentiation. The knockdown of SETDB1 removes H3K9me3 and facilitates adipogenesis. Recently, two other H3K9me2/me3 methyltransferases suppressor of variegation 3–9 homolog 1 (SUV39H1/KMT1A) and 2 (SUV39H2/KMT1B) displayed an increase in expression during 3T3L-1 adipogenesis [68]. The knockdown of both of these proteins inhibited adipogenesis, while overexpression promoted adipogenesis. Mechanistically, SUV39H1 inhibits the *Wnt10a* gene via H3k9 methylation expression and its interaction with DNA methyltransferase DNMT1. 

Besides H3K4, H3K9 methylations, H3K27 methylation, and H3k36 methylation also play an essential role in regulating adipogenesis. Histone lysine methyltransferase, enhancer of zeste homolog 2 (EZH2/KMT6) and its H3K27me3 activity are required for adipogenesis. EZH2 promotes adipogenesis by directly repressing *Wnt1*, *6*, *10a*, and *10b* genes [69]. Loss of EZH2 eliminates H3K27me3 on *Wnt* promoters and inhibits adipogenesis of preadipocytes. H3.3K36M mutation lacking H3K36 methylation in preadipocytes inhibited adipogenesis by elevating the levels of H3K27me^3^ on genes like *Cebpα* and *Pparγ*. The depletion of H3K36 methyltransferase NSD2 (KMT3G) displayed a similar effect to H3.3K36M on adipogenesis, suggesting that H3K36 methylation regulates adipogenesis positively [70]. Furthermore, a recent study revealed the importance of H3K20 methyltransferases SUV420H1 (KMT5B) and SUV420H2 (KMT5C) in regulating adipogenesis and PPARγ. Mice lacking both SUV420H1 and SUV420H2 in *Myf5* lineage displayed improved mitochondrial respiration and glucose tolerance, which suggests that SUV420H proteins inhibit the thermogenesis program in mice [71]. These mice also displayed increased browning in eWAT. In contrast, another study showed that the lentivirus-mediated knockdown of SUV420H2 inhibited the thermogenic gene program in BAT and WAT [72]. Interestingly, adipocyte-specific KO mice of SUV420H2 have significantly less UCP1 expression in WAT, but not in BAT when fed with a chow diet. Additionally, the expression of *Suv420h2* is significantly induced by β3-AR signaling in both white and brown fat. Furthermore, these SUV420H2 knockout mice are prone to high fat-induced obesity, suggesting the crucial role of H3K20 methylation in regulating WAT browning. 

Protein arginine methyl transferases that mediate histone arginine methylation (PRMTs) also play an important role in adipogenesis. PRMT5 has been shown to promote adipogenesis by inducing the dimethylation of histones and also is required for recruiting Brg1-based ATP-dependent SWI/SNF chromatin remodeling enzymes to PPARγ2-based adipogenic promoters [73]. Another protein arginine methyl transferase, PRMT7, does not affect the adipogenesis of C3H10T1/2 cells and NIH3T3 fibroblasts [73]. In contrast to PRMT5, PRMT6 is reported to inhibit adipogenesis when overexpressed and promoted adipogenesis when silencing its expression [74]. Mechanistically, Prmt6 interacts with PPARγ and represses adipogenic genes by inducing repressive arginine trimethylation. In summary, MLL3, MLL4, SUV39H1, SUV39H2, EZH2, and NSD2 are reported to promote adipogenesis while EHMT1 and SUV420H2 promote white adipocyte browning. MLL3 and MLL4 are important for brown adipocytes function, but their specific role in WAT browning is unclear.

## 5. Histone Demethylases

Histone demethylases carry out histone demethylation reactions. Silencing of H3K4me1/me2 demethylase lysine-specific demethylase 1 (LSD1/KDM1A) in 3T3L1 cells significantly abrogated adipogenesis with decreased H3K4me2 and increased H3K9me2 [75]. Additionally, cold and β3-adrenergic stimulation elevates LSD1, and this increased LSD1 level promotes mitochondrial activity in WAT. Transgenic mice with elevated LSD1 expression displayed decreased weight gain when fed an HFD, and ChIP experiments further confirmed that LSD1 directly stimulates the genes associated with oxidative phosphorylation [76]. Moreover, the conversion of beige adipocytes into white adipocytes was associate with decreased LSD1 expression during aging, suggest the dynamic role of LSD1 in WAT browning. Additionally, the adipocyte-specific expression of LSD1 conserves beige adipocytes in mice during aging [77]. Additionally, Lysine-specific demethylase 2 (LSD2/KDM1B) knockdown in WAT cells showed a drastic increase in myogenic genes, while in BAT cells, it showed decreased expression of brown specific genes, indicating LSD2 might play a role in maintaining WAT cell identity [78]. The lysine demethylase 5 (LSD5/KDM5) family histone demethylases delete H3K4me3. Genome-wide studies revealed that these proteins regulate cell cycle and mitotic clonal expansion in 3T3L-1 cells and brown preadipocytes. Loss of KDM5 proteins blocks preadipocytes’ differentiation into mature adipocytes by interfering with the cell cycle and cell proliferation [79]. In 3T3L-1 cells, CEBPβ has shown to transactivate LSD5A (KDM5A) to further downregulate Wnt6, a negative regulator of adipogenesis [80]. LSD5A deletion is also associated with the restoration of mitochondrial function by regulating PGC1α protein in pRB-negative breast cancer cell lines, indicating its potential role in thermogenesis [81].

KDM3A (JMJD1A) has been induced by β3-adrenergic stimulation and directly regulates *pparα* and *ucp1* genes in brown adipocytes [82,83,84]. Additionally, KDM3A is recruited to the PPAR target gene promoters and demethylates H3K9me2 to facilitate the binding of coactivators. Moreover, loss of KDM3A in mice results in abnormal fat accumulation in WAT, obesity, and hyperlipidemia, suggesting that KDM3A is crucial for WAT browning. KDM3C (JMJD1C) and KDM4B (JMJD2B) in 3T3L-1 cells promoted adipogenesis by demethylating H3K9me2/me3 to induce adipocyte-specific TFs [85,86]. The specific role of KDM3Cand KDM4B in WAT browning is yet to be examined. Demethylation of H3K9me3 by KDM4D (JMJD2D) and its physical interaction with the MLL1 complex is crucial for the induction of PPARγ and CEBPα during adipogenesis of C3H10T1/2 cells [87]. KDM4A (JMJD2A) removes H3K9me^3^, while KDM7A removes H3K9me2 and H3K27me2 on *sfrp4* and *cebpα* gene promoters to promote the adipogenesis of stromal cells [88,89]. In tamoxifen-induced conditional knockout, loss of KDM7C (PHD finger protein 2/PHF2) abated adipogenesis [90]. Mechanistically, KDM7C interacts with CEBPα and demethylates H3K9me2 on CEBP target gene promoters. A recent report revealed that H3K27me^3^ marks BAT genes but not common WAT genes, and their demethylation by KDM6B (JMJD3) is required for BAT-selective gene expression and WAT browning [91]. Transgenic mice expressing the *Kdm7c* gene displayed increased UCP1 in WAT, indicating that KDM7C promotes WAT browning. Another H3K27 demethylase, KDM6A (ubiquitously transcribed tetratricopeptide repeat, X chromosome/UTX), positively regulates brown fat thermogenesis. Upon cold exposure, the induction of UCP1 is associated with UTX expression in both BAT and WAT [92]. In summary, despite the site that they demethylate, most of the histone demethylases promote adipogenesis, and KDM1A, KDM3A, KDM6A, and KDM6B are reported to promote white adipocyte browning. All the histone-modifying enzymes that play essential role in white adipocytes browning are listed in Table 1.

## 6. DNA Methyltransferases and Demethylases

DNA methylation involves converting cytosine to 5-methyl cytosine, resulting in gene repression either by recruiting repressive factors or by inhibiting TF binding. DNA methylation altered during development, and differentiated cells develop a stable and unique DNA methylation pattern that defines tissue-specific transcription [93]. The role of DNA methylation in adipogenesis was initially identified in the studies where clones of swiss 3T3 cells showed enhanced differentiation into adipocytes without any external stimulus when treated with DNA methylation inhibitor 5-azacytidine [94]. Additionally, the same treatment commits MSCs to the adipocyte lineage. During 3T3L-1 adipogenesis, DNA methylation at CpG sites has affected GLUT4 promoter activity and expression [95]. One of the nuclear receptor corepressors, receptor-interacting protein 140 (RIP140), directs the assembly of DNA methyltransferases on the *ucp1* enhancer and contributes to its gene repression forming CpG sites in white adipocytes [96]. Another study showed that *ucp1* expression in BAT was associated with decreased CpG DNA methylation at its enhancer and increased H3K4me3 on the *ucp1* promoter in response to cold exposure [97]. Hypocaloric diet-induced weight loss in humans was also associated with altered DNA methylation [98]. Notably, reduced representation bisulfite sequencing and RNA sequencing experiments revealed differential DNA methylation between white and brown adipocyte lineages. Blocking DNA methylation by 5-azacytindine, increased Hox gene expression, especially the *hoxc10* gene, a negative regulator of brown fat, indicating the positive role of DNA methylation in BAT [99]. 5-azadeoxycytidine decreased the proliferation and adipocyte differentiation of human MSCs [100]. Dexamethasone also favors adipocyte differentiation of bone marrow stromal cells by inhibiting the cebpα promoter’s hypermethylation, suggesting that these chemical compounds affect adipocyte differentiation mostly by altering DNA methylation patterns [101]. Whole-body insulin resistance, a hallmark of type 2 diabetes, was also associated with genome-wide DNA methylation patterns [102].

DNA establishes de novo DNA methylation (cytosine-5)-methyltransferase, Dnmt3a, and Dnmt3b, while it is sustained by Dnmt1 [103,104]. Adipose-specific expression of Dnmt3a in mice did not significantly affect the DNA methylation, while gene expression of inflammatory cytokines was higher, suggesting that Dnmt3a might regulate obesity-related inflammation in mice [105]. Mice with adipose-specific deletion of Dnmt3a are guarded against diet-induced insulin resistance through upregulated *fgf21* expression, indicating that FGF21, as a crucial regulator, is affected by Dnmt3a in adipocytes [106]. In human myotubes, the *pgc1α* promoter is hypermethylated in non-CpG sites, and silencing of Dnmt3b prevented this hypermethylation [107]. The expression of another DNA methyltransferase, Dnmt1, is reported to be induced during 3T3L-1 adipocyte differentiation, but its silencing accelerated adipocyte differentiation. Additionally, it alters H3K9 methylation during adipogenesis [108]. Dnmt1 deletion enhanced lipid accumulation by promoting SREBP1C expression during adipogenesis [103]. Additionally, Dnmt1 has been shown to be crucial for inhibiting myogenic genes in brown adipocytes [109]. A very recent report showed that cold or beige adipogenesis inducers suppress the expression of DNA demethylase ten-eleven translocation 1 (TET1) in subcutaneous white adipose tissue. Adipose selective knockout of *Tet1* displayed energy expenditure and protected mice against diet-induced obesity [110]. However, it has also been shown that TET1-mediated suppression of thermogenic genes is mediated through HDAC1, but not due to its own DNA demethylase activity.

## 7. Transcriptional Factors of White Adipocytes and Browning

PPARs, CCAAT/Enhancer Binding Proteins (CEBPs), kruppel-like factors (KLFs), and signal transducer and activator of transcriptions (STATs) are the prominent family of TFs that positively regulate adipogenesis [111,112,113]. PPARγ is established as a master transcriptional regulator of adipogenesis as it can be sufficient alone to induce adipocyte differentiation from fibroblasts and muscle cells [114]. CEBPβ and CEBPδ are reported to be induced during initial hours of adipocyte differentiation, and they cooperate with other TFs like the glucocorticoid receptor, STAT5A. This first wave of TFs creates hotspots that are replaced by the PPARγ and CEBPα [115]. Genome-wide analysis revealed that the PPARγ and CEBPα bind in the vicinity of each other to regulate adipogenesis [116]. The key adipogenic TFs common for both white and thermogenic adipocytes were elaborately reviewed [111,115,117]. Interestingly, earlier studies revealed that PPARγ could activate the *ucp1* promoter only in brown adipocytes but not in fibroblasts, indicating the existence of a brown adipocyte-specific cofactor named PGC1α. PRDM16 was another coregulator of PPARγ reported to be essential for the thermogenesis program in both BAT and WAT [118,119]. BAT-specific TFs that might play a role in WAT browning have previously been reviewed elaborately [120,121]. This review mainly focused on the recent literature about TFs that are either crucial for white adipocyte differentiation or their browning.

TLE3: TLE3 belongs to the transducing-like enhancer of split (TLE) protein family of transcriptional coregulators. Earlier, a high throughput cDNA screen identified TLE3 as a PPARγ [122]. Additionally, overexpression of TLE3 was shown to mimic the PPARγ agonist effect in regulating insulin resistance. Furthermore, TLE3 is emphasized as a white adipose-selective cofactor for PPARγ that counteracts thermogenic cofactor PRDM16 and prevents WAT browning. Additionally, when overexpressed in brown fat, it suppresses brown genes and induces white adipose genes, suggesting the importance of TLE3 in maintaining white adipocyte cell identity [123]. Notably, mice lacking TLE3 in adipose tissue showed increased thermogenesis in inguinal white adipose tissue, suggesting that the cell-type-specific recruitment of cofactor by PPARγ defines the white vs. brown cell identity. Additionally, TLE3 is responsible for the age-induced decline in mitochondrial oxidative phosphorylation by inhibiting the recruitment of EBF2 to mitochondrial gene promoters [124].

ZFP423: Previous research showed that the C2H2 Zinc finger protein ZFP423 (Zinc finger protein 423) is necessary for preadipocyte commitment as its overexpression in non-adipogenic fibroblasts induced PPARγ and adipocyte differentiation [125]. Additionally, ZFP423 is crucial for preadipocyte commitment by co-activating Smad proteins in the bone morphogenic protein signaling pathway. Doxycycline-induced deletion of *zfp423* in mature adipocytes of adult mice and β-adrenergic stimulation initiated the conversion of mature white adipocytes to beige adipocytes, indicating that ZFP423 suppresses the thermogenic transcription program in fully differentiated inguinal white adipocytes [126]. Mechanistically, ZFP423 inhibits the ability of Ebf2 to activate PRDM16.

ZFP238: A recent study indicated that ZFP238 (zinc finger protein 238) is a regulator of the thermogenic program in white adipocytes. Mice with adipose-specific ablation of *zfp238* displayed decreased oxygen consumption, energy expenditure, and UCP1 expression in response to cold or β3 agonists [127]. UCP1 induction was abolished in the absence of ZFP238 when 3T3L-1 cells were exposed to cold or forskolin, but the deletion of both *zfp238* and *foxo1*-rescued UCP1 expression suggests that ZFP238 acts as a positive regulator of white adipocyte thermogenesis by inhibiting the Foxo1 protein. Moreover, the physical interaction between ZFP238 and FOXO1 has been identified using a yeast two-hybrid screen of the 3T3L-1 cDNA library, and this interaction inhibits FOXO1 transcriptional activity, thereby regulating thermogenesis.

ATF7: ATF7 (Activating transcription factor 7) belongs to the ATF2 subfamily of TFs, and it represses gene expression by recruiting histone methyltransferases to gene promoters. ATF7 knockout mice displayed decreased adipose tissue mass, body weight, and resistance to diet-induced obesity [128]. Interestingly, these knockout mice exhibited comparable energy expenditure to wildtype littermates but displayed increased energy expenditure when fed an HFD. Preadipocytes from ATF7 knockout mice displayed reduced differentiation into adipocytes [129]. Conversely, ATF7 is also reported to be required for adipogenesis by repressing interferon-stimulated genes by recruiting histone methyltransferase G9a. Mechanistically, ATF7 induces repressive histone modification H3K9me2 on the STAT1 promoter to regulate inflammatory genes. Additionally, loss of ATF7 promoted browning of inguinal WAT in mice, while its overexpression inhibited thermogenesis in white adipocytes, suggesting that ATF7 is crucial for maintaining white adipocyte cell identity. Additionally, ATF7 also represses UCP1 expression by physically interacting with CEBPβ to induce H3K9me2 on the *ucp1* enhancer.

Id1: Inhibitor of differentiation 1 (Id1) is a helix-loop-helix TF without a DNA binding domain, and it plays a crucial role in cellular proliferation and differentiation. Mice with adipose-specific overexpression of *Id1* displayed reduced energy expenditure, increased body weight, and fat mass [130]. These mice are also prone to HFD-induced obesity. Mechanistically, Id1 binds to PGC1α and inhibits its transcriptional activity in BAT. Additionally, loss of Id1in mice significantly upregulated WAT browning when exposed to cold. This indicates that Id1 specifically inhibits BAT-associated thermogenesis in mice, while its loss promoted WAT browning, showing how crucial Id1 might be towards maintaining white adipocyte identity.

ERα: Obese women have lower levels of estrogen receptor α (ERα) compared to non-obese women, which indicates the association between ERα with obesity [131]. Later, *ESR1/esr1,* the gene encoding ERα, was also reported to inversely correlate to fat mass and insulin sensitivity in both humans and mice [132]. Studies on WAT-specific deletion of *esr1* in mice revealed the inverse correlation of esr1 with mitochondrial DNA copy number, and this phenomenon was also observed in humans. RNA seq analysis of epididymal WAT in adipose-specific *esr1* knockout mice showed a marked reduction in the expression of *Pgc1b*, *Nrf1*, *Polg1* (encodes the catalytic subunit of polymerase γ), and *Polrmt* (encodes primary mitochondrial RNA polymerase). It was identified that ERα directly binds to the *Polg1* promoter and regulates its expression and mtDNA copy number. ER agonist pyrazole triol also induced browning in 3T3L-1 white preadipocytes, and ERα knockdown reduced the ability of pyrazole triol-induced browning. In addition, overexpression of ERα in its knockout mice also induced WAT browning [133]. Furthermore, ERβ agonist LY3201 treatment also induced subcutaneous adipose tissue browning, indicating the importance of estrogen receptors in white adipocyte browning [134].

IRX3: Iroquois homeobox protein 3 (IRX3) belongs to the Iroquois family of homeobox TFs, shown to participate in the development of different tissues [135]. The exciting role of IRX3 with obesity came into light with the genome-wide association studies, which identified the direct interaction of IRX3 gene promoter with obesity-associated *FTO* (fat mass and obesity-associated) gene regions [136]. IRX3 KO mice displayed a reduction in body weight by 25–30%, suggesting the direct role of IRX3 in controlling body mass index. Cold exposure induced *Irx3* mRNA expression and is correlated with *Ucp1* expression. Furthermore, knockdown of *Irx3* in white preadipocytes significantly abrogated the expression of brown adipocyte markers such as *Prdm16*, *Ucp1*, *Cidea*, and *Pgc1α* when treated with β3-AR agonist CL-316,243. Interestingly, it has been identified that IRX3 can bind directly to the *Ucp1* promoter, and induces its expression, indicating the vital role of IRX3 in adipogenesis and browning.

NKX1-2: NK1 homeobox 2, a TF that belongs to the NKX family of proteins, is shown to be induced during 3T3L-1 adipocyte differentiation [137]. Additionally, shRNA-mediated knockdown of NKX1-2 in 3T3L-1 cells or in-ear MSCs wholly abrogated adipocyte differentiation. Mechanistically, NKX1-2 promotes adipocyte differentiation by inhibiting COUP-TF II expression. Another protein from the NKX family, NKX2-1, inhibited adipogenesis when overexpressed in 3T3L-1 cells and in thyroid cancer cells expressing the PAX8-PPARγ fusion oncogene, observed by a reduced number of lipid droplets and decreased expression of adipocyte marker genes [138].

HLX: An H2.0-like homeobox (HLX) is a TF reported to be expressed in inguinal WAT (iWAT) and BAT. The β3-AR agonist CL-316,243 or forskolin induced HLX protein but not its mRNA [139]. Additional experiments revealed that CL-316,243 suppressed the translational inhibitor 4E-BP, thereby increasing the translational efficiency of *hlx* mRNA. Experiments with *hlx* heterozygous mice or mice with specific knockdown of Hlx by injecting adenovirus-expressing shRNA to fat pads displayed decreased expression of *ucp1* and mitochondrial genes. Mice expressing the *hlx* transgene displayed increased browning of iWAT, suggesting that Hlx positively regulates thermogenesis [139]. Mechanistically, Hlx physically interacts with PRDM16 and functions as a coactivator to regulate UCP1 expression by directly binding to its promoter.

BCL6: B-cell lymphoma 6 (BCL6) is enriched in preadipose vs. non-preadipose fibroblasts and displayed increased upregulation in the early stage of adipogenesis. The knockdown of BCL6 in C3H10T1/2 cells inhibited adipogenesis, while overexpression enhanced adipogenesis [140]. Adipocyte-specific knockout of BCL6 in mice possessed increased iWAT and displayed enhanced insulin sensitivity [141]. Unlike other TFs reported earlier to play a role in thermogenesis upon cold adaptation, BCL6 is crucial for maintaining brown adipocytes’ cell identity during dormancy. Loss of BCL6 had a profound effect on brown fat competence when mice were bred at thermoneutrality (30 ℃) and then challenged to cold at 10 °C [142]. Bcl6^f/f^Ucp1Cre and Bcl6^f/f^Myf5Cre mice exposed to cold from thermoneutrality resulted in hypothermia, and oxygen consumption was also reduced by 40%. Notably, brown adipocyte-specific enhancers displayed decreased H3K27ac, and white adipose-specific enhancers displayed increased acetylation in the BAT of Bcl6^f/f^Ucp1Cre mice, which resulted in increased white adipose-specific genes in brown adipocytes upon cold exposure from thermoneutrality. These results suggest that BCL6 is crucial for maintaining cell identity in adipocytes.

ATF3: Activating transcription factor 3-3 (ATF3) is a stress-inducible gene shown to regulate adiponectin expression in 3T3L-1 adipocytes [143]. Lentiviral-mediated overexpression of ATF3 inhibited differentiation and lipid accumulation in 3T3L-1 adipocytes by inhibiting *cebpα* and *pparγ* promoter activity and expression [144,145]. Moreover, ATF3 has been shown to contribute to mitochondrial dysfunction associated with obesity in mice, and its overexpression in 3T3L-1 cells also decreased the expression of mitochondrial genes [146]. HFD-fed *ATF3^−/−^* mice exhibited aggravated obesity and metabolic dysfunction [147]. Adenoviral-mediated overexpression of ATF3 in *ATF3^−/−^* mice significantly improved glucose tolerance and insulin sensitivity. Interestingly, ATF3 overexpression suppressed the expression of genes associated with white adipogenesis but increased the expression of genes *ucp1* and *pgc1α* in 3T3L-1 cells, suggesting the role of ATF3 in adipocyte browning. Mechanistically, ATF3 suppresses ChREBP-SCD1 signaling by directly binding to the *chrebp* gene promoter and regulates white adipocyte browning. 

**Figure 3:**
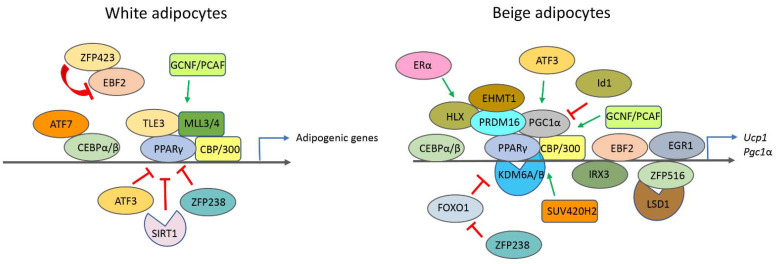
Transcriptional factors and histone-modifying enzymes in regulating white and beige adipocytes gene promoters.

EGR1: Zinc figure transcription factor ZNF268, also called EGR1 (early growth response protein 1) or NGFI-A (nerve growth factor-induced protein A), has been shown to inhibit white adipocyte browning by directly repressing *ucp1* promoter activity [148]. Moreover, loss of *Egr1* was enough to induce brown-like adipocytes from mouse embryonic stem cells, and *Egr1* mutant mice also displayed increased WAT browning, suggesting that *Egr1* is crucial for maintaining white adipocyte cell identity [149]. A summary of the transcriptional regulators crucial for white adipocytes and beige adipocytes is represented in Figure 3.

## 8. miRNA

Micro RNA (miRNAs) are small non-coding RNAs that regulate gene expression at the post transcriptional level [150]. miRNAs have previously been shown to regulate a wide range of biological processes including adipogenesis. MiR-32 is a BAT-specific super enhancer-associated miRNA that upregulates upon cold exposure. Inhibiting miR-32 compromised WAT browning and BAT activation [151]. Mechanistically, miR-32 inhibits the *Tob1* gene, thereby activating P38 MAP kinase and driving FGF21 secretion by BAT, which further induces WAT browning. In a recent study, tryptophan-derived metabolites produced by gut microbiota have been shown to induce miR-181 in white adipocytes and promote insulin sensitivity and energy expenditure in mice [152]. Additionally, loss of the gut microbiota–miR181 axis is required for the development of obesity in mice. Deletion of miR-26a, 26b, and 26c loci from mice resulted in significant expansion of adipose tissue in adult mice and this abrupt expansion was attributed to increased proliferation of adipocyte progenitor cells [153]. Overexpression of miR-26a protected mice from HFD-induced obesity. Several miRNAs that play a role in WAT and BAT function have already been reviewed elsewhere [154,155]. miR-133, miR-27, and miR-150 directly repress PRDM16 and other browning genes, while miR-34a targets FGF21 to inhibit WAT browning [156,157,158,159]. miR-196a suppresses the expression of the white specific *Hoxc8* gene to induce WAT browning [160] (22545021). miRNA-155 represses CEBPβ to abrogate adipogenesis while its inhibition promotes browning in both WAT and BAT [161]. miR-455 is induced by cold and BMP7, when overexpressed, markedly increased WAT browning by activating AMPKα1 [162]. miR-30b/c promoted WAT browning by inhibiting RIP140, a nuclear receptor corepressor that inhibits browning in WAT [163]. miRNA Let-7i-5p and miR-125b-5p overexpression inhibited browning of WAT, suggesting that these miRNAs are crucial for white adipocyte identity [164,165].

## 9. Concluding Remarks

Recent research work revealed that cold-induced beige adipocytes display epigenetic modifications that are quite similar to brown adipocytes. In contrast, warm conditions induce epigenetic modifications in beige adipocytes that are quite similar to white adipocytes, indicate the dynamic role of epigenetic regulators in this process [12]. Earlier findings also revealed the role of several histone modifying enzymes in white adipocytes and beige adipocytes (Table 1). The epigenetic regulators reported in this review are shown to induce either differentiation or browning of white adipocytes (Figure 1 and Figure 2). With the recent identification of beige preadipocytes in WAT, how these epigenetic regulators affect beige preadipocyte identity and differentiation still needs to be investigated. Additionally, several of these regulators are shown to regulate adipogenesis just based on studies with cell lines such as 3T3L-1 cells or C3H10T1/2. Hence, their exact role in white adipocyte cell identity or browning is yet be investigated. We hope that the information provided in this literature review acts as point of reference and is useful for researchers working in the field related to metabolic abnormalities.

## Figures and Tables

**Table 1:** Histone modifying enzymes in white adipocyte differentiation and browning.

Histone Modification	Effector	Epigenetic Mark	Role in Adipogenesis or White Browning	Reference
Histone acetylation	CBP and P300	H3K27ac	Promote adipogenesis	[21]
		H3K27ac	Promotes white adipocyte browning	[12]
	GCN5 and PCAF	H3K9ac	Promotes WAT browning and brown preadipocyte differentiation	[24,25]
Histone deacetylation	HDAC1 and HDAC2	H3K27ac	Promotes adipogenesis	[33]
	HDAC3	H3K27ac	Promotes adipogenesis and inhibits WAT browning	[31]
	HDAC9	H3K27ac	Inhibits adipogenesis	[35]
	HDAC11	H3K27ac	Inhibits white adipocyte browning	[32]
	SIRT1	H3K9ac, H4K16ac	Inhibits adipogenesis but promotes white adipocyte browning	[41,42,43]
	SIRT2	H3K9ac	Inhibits adipogenesis and white adipocyte browning	[44]
	SIRT3	H3K9ac	Promotes white adipocyte browning	[47]
	SIRT5	H3K9ac	Inhibits white adipocyte browning	[49]
	SIRT6	H3K9ac	Promotes adipogenesis	[51]
	SIRT7	H3K9ac, H4K16ac	Inhibits adipogenesis	[52]
Histone Methylation	MLL3	H3K4me3	Promotes adipogenesis	[59]
	MLL4	H3K4me3	Promotes adipogenesis	[60,61]
	EHMT1	H3K9me2/3	Promotes white adipocyte browning	[65]
	G9A	H3K9me2	Inhibits adipogenesis	[66]
	SETDB1	H3K9me3	Inhibits adipogenesis	[67]
	SUV39H1 SUV39H2	>H3K9me2/me3	Promotes adipogenesis	[68]
	EZH2	H3K27me3	Promotes adipogenesis	[69]
	NSD2	H3K36me3	Promotes adipogenesis	[70]
	>SUV420H2	H3K20me3	Promotes white adipocyte browning	[72]
	PRMT5	H3R8me2	Promotes adipogenesis	[73]
	PRMT6	H3R2me2	Inhibits adipogenesis	[74]
Histone Demethylation	KDM1A	H3K4me2	Promotes adipogenesis and white adipocyte browning	[77]
	KDM1B	H3K4me2	Promotes adipogenesis	[78]
	KDM5A	H3K4me3	Promotes adipogenesis	[80]
	KDM3A	H3K9me	Promotes white adipocyte browning	[82,83,84]
	KDM3C	H3K9me	Promotes adipogenesis	[85]
	KDM4B	H3K9me	Promotes adipogenesis	[86]
	KDM4D	H3K9me3	Promotes adipogenesis	[87]
	KDM4A	H3K9me3	Promotes adipogenesis	[88]
	KDM7A	H3K9me2, H3K27me2	Promotes adipogenesis	[89]
	KDM7C	H3K9me2	Promotes adipogenesis	[90]
	KDM6B	H3k27me3	Promotes white adipocyte browning	[91]
	KDM6A	H3k27me3	Promotes white adipocyte browning	[92]
